# Clinical Significance of Fronto-Temporal Gray Matter Atrophy in Executive Dysfunction in Patients with Chronic Kidney Disease: The VCOHP Study

**DOI:** 10.1371/journal.pone.0143706

**Published:** 2015-12-03

**Authors:** Kazuhiko Tsuruya, Hisako Yoshida, Naoki Haruyama, Kiichiro Fujisaki, Hideki Hirakata, Takanari Kitazono

**Affiliations:** 1 Department of Integrated Therapy for Chronic Kidney Disease, Graduate School of Medical Sciences, Kyushu University, Fukuoka, Japan; 2 Department of Medicine and Clinical Science, Graduate School of Medical Sciences, Kyushu University, Fukuoka, Japan; 3 Division of Nephrology and Dialysis Center, Japanese Red Cross Fukuoka Hospital, Fukuoka, Japan; Brighton and Sussex Medical School, UNITED KINGDOM

## Abstract

**Background & Objectives:**

It is well known that cognitive impairment in patients with chronic kidney disease (CKD) is characterized by executive dysfunction, rather than memory dysfunction, although the precise mechanism of this remains to be elucidated. The purpose of the present study is to examine the correlation between gray matter volume (GMV) and executive function in CKD patients.

**Design, Setting, Participants, Measurements:**

This cross-sectional study recruited 95 patients with non-dialysis-dependent CKD (NDD-CKD) with no history of cerebrovascular disease, who underwent brain magnetic resonance imaging (MRI) and Trail Making Test (TMT) in the VCOHP Study. The subjects underwent brain MRI and TMT part A (TMT-A) and part B (TMT-B). The segmentation algorithm from Statistical Parametric Mapping 8 software was applied to every T1-weighted MRI scan to extract tissue maps corresponding to gray matter, white matter, and cerebrospinal fluid. GMV was normalized by dividing by the total intracranial volume, calculated by adding GMV, white matter volume, and cerebrospinal fluid space volume. Then, normalized whole-brain GMV was divided into four categories of brain lobes; frontal, parietal, temporal, and occipital. We assessed the correlation between normalized GMV and TMT using multivariable regression analysis.

**Results:**

Normalized whole-brain GMV was significantly inversely correlated to the scores of TMT-A, TMT-B, and ΔTMT (TMT-B minus TMT-A). These correlations remained significant even after adjusting for relevant confounding factors. Normalized frontal and temporal GMV, but not parietal and occipital GMV, were significantly inversely correlated with TMT-A, TMT-B, and ΔTMT using multivariable regression analysis.

**Conclusions:**

The present study demonstrates the correlation between normalized GMV, especially in the frontal and temporal lobes, and executive function, suggesting that fronto-temporal gray matter atrophy might contribute to executive dysfunction in NDD-CKD.

## Introduction

Recently, accumulating evidence has been published on cognitive impairment in patients with chronic kidney disease (CKD). It has become clear that the prevalence of cognitive impairment is increased in not only dialysis patients, but also in non-dialysis-dependent CKD (NDD-CKD) patients [1,2]. The symptoms and characteristics of cognitive impairment in patients with CKD are characterized by vascular cognitive impairment, believed to be caused by damaged blood vessels in the brain, or cerebrovascular disease, rather than Alzheimer-type dementia [3,4]. Frontal lobe dysfunction, characterized by executive dysfunctions such as disorganization, loss of mental flexibility, impaired problem solving, decreased insight, and impaired working memory, is a feature of vascular dementia or vascular cognitive impairment [5]. A recent study reported by Yao et al. [6] provided further evidence that CKD may be an independent risk factor for frontal, rather than global, cognitive dysfunction, suggesting that CKD acts as a vascular factor.

On the other hand, it has been reported that the progression of brain atrophy is rapid in CKD patients, especially in patients on hemodialysis (HD) [7–9]. A recent study reported by Yakushiji et al. [10] suggests that patients with a glomerular filtration rate (GFR) of less than 60 mL/min/1.73 m^2^ have a higher risk of cortical atrophy than those with normal renal function.

The Trail Making Test (TMT) is a neuropsychological test designed to assess a subject’s visual attention and task-switching ability and is a widely used and reliable measure of frontal lobe executive functions [11,12]. Results from a recent study [13] suggest that TMT-A requires mainly visuoperceptual abilities whilst TMT-B reflects primarily working memory and secondarily task-switching ability. The difference score B–A (ΔTMT) minimizes visuoperceptual and working memory demands, providing a relatively pure indicator of executive control abilities.

Some evidence has been reported that brain atrophy correlates with cognitive impairment in various conditions [14–19], whereas in CKD, there is only one report of such a correlation in end-stage renal disease (ESRD) patients [20], but not in NDD-CKD patients. Thus, to elucidate the impact of brain atrophy on cognitive impairment, we examined the correlation between normalized gray matter volume (GMV) and executive function in patients with CKD stages 3–5 in the present study.

## Materials and Methods

### Ethics statement

This study was approved by the Institutional Review Board of Kyushu University (#23–112), registered in the UMIN clinical trial registry as the VCOHP Study (UMIN000001589), and conducted in accordance with Declaration of Helsinki. All participants provided their written informed consent to participate in this study.

### Subjects

Since December 2008, to investigate the degree of progression of cerebro- and cardiovascular complications in NDD-CKD, HD, and peritoneal dialysis (PD) patients, we have conducted an observational study named the Observational Study on Cerebro- and Cardiovascular Complication in Non-dialysis-dependent, Hemodialysis, and Peritoneal Dialysis Patients with Chronic Kidney Disease (VCOHP Study). Inclusion criteria are as follows: (1) patients aged 20–80 years at the time of entry into the study; and (2) NDD-CKD patients whose estimated glomerular filtration rate (eGFR) was less than 60 mL/min/1.73 m^2^ irrespective of urinalysis findings (CKD stages 3–5) or patients with ESRD on either HD or PD, who started dialysis within 2 years of study entry. Exclusion criteria are as follows: (1) pregnant women, or women who have the possibility of pregnancy, (2) patients who have previously received another dialysis therapy for longer than 3 months, (3) patients who have previously undergone renal transplantation, and (4) patients who have a previous history of brain injury, such as symptomatic stroke, traumatic brain injury, brain tumor, or any neuropsychiatric disease.

By July 2014, 212 patients (34 HD patients, 72 PD patients, and 106 NDD-CKD patients) were entered into the VCOHP Study. The present study included only the NDD-CKD patients because brain atrophy in dialysis patients was significantly more severe than in NDD-CKD and it is thought that evaluation of the brain atrophy might be influenced largely by the status of dialysis requirement [21]. Of the 106 NDD-CKD patients, images of magnetic resonance imaging (MRI) were not available because of the poor quality of the images in two patients and TMT was not performed in nine patients. Thus, the remaining 95 patients were included in the present study. The clinical characteristics and the laboratory data for these patients are shown in Table 1.

**Table 1:** Clinical Characteristics and Laboratory Data of All Participants According to CKD Stages. Values for categorical variables are given as number (percentage); values for continuous variables are given as mean ± standard deviation or median (interquartile range). Abbreviations: eGFR, estimated glomerular filtration rate; ESA, erythropoiesis-stimulating agent; HDL, high-density lipoprotein; LDL, low-density lipoprotein; NT-proBNP, N-terminal pro-brain natriuretic peptide; PTH, parathyroid hormone; RAAS, renin-angiotensin-aldosterone system; UPCR, urinary protein to creatinine ratio.

	All participants (*n* = 95)	CKD stage 3a (*n* = 34)	CKD stage 3b (*n* = 26)	CKD stages 4+5 (*n* = 35)	*P* for trend
**Age [years]**	62 ± 11	63 ± 11	60 ± 11	64 ± 12	0.750
**Sex, male, *n* (%)**	49 (52)	14 (42)	13 (50)	22 (63)	0.071
**Diabetes mellitus, *n* (%)**	28 (30)	6 (18)	5 (19)	17 (49)	0.005
**Smoking habits, current/past, *n* (%)**	9 (9) / 42 (44)	1 (3) / 9 (26)	2 (8) / 12 (46)	6 (17) / 21 (60)	0.002 *
**Daily alcohol consumption, *n* (%)**	44 (46)	12 (35.3)	16 (61.5)	16 (45.7)	0.393
**Previous history of CVD, *n* (%)**	11 (12)	2 (6)	2 (8)	7 (20)	0.066
**Education of more than 12 years, *n* (%)**	37 (39)	9 (27)	14 (54)	14 (40)	0.255
**Body mass index [kg/m** ^**2**^ **]**	24.0 ± 4.0	24.5 ± 4.1	23.9 ± 3.6	23.7 ± 4.2	0.704
**Systolic blood pressure [mmHg]**	135 ± 17	135 ± 17	134 ± 18	136 ± 17	0.878
**Diastolic blood pressure [mmHg]**	81 ± 11	83 ± 12	81 ± 10	79 ± 11	0.384
**Medication**					
**Use of RAAS inhibitors, *n* (%)**	79 (83)	25 (74)	22 (85)	32 (91)	0.047
**Use of calcium antagonists, *n* (%)**	55 (58)	17 (50)	12 (46)	26 (74)	0.040
**Use of statins, *n* (%)**	49 (52)	17 (50)	14 (54)	18 (51)	0.907
**Use of ESAs, *n* (%)**	13 (14)	0 (0)	0 (0)	13 (100)	<0.001
**Laboratory data**					
**Total protein [g/dL]**	6.8 ± 0.5	6.8 ± 0.4	6.7 ± 0.4	6.8 ± 0.7	0.817
**Albumin [g/dL]**	3.9 ± 0.4	4.1 ± 0.3	3.9 ± 0.4	3.8 ± 0.5	0.021
**Serum urea nitrogen [mg/dL]**	30.3 ± 16.4	20.2 ± 4.0	23.5 ± 6.4	45.3 ± 18.0	<0.001
**Creatinine [mg/dL]**	1.72 ± 1.15	0.97 ± 0.21	1.30 ± 0.18	2.75 ± 1.35	<0.001
**Uric acid [mg/dL]**	6.5 ± 1.5	5.8 ± 1.4	6.7 ± 1.6	7.0 ± 1.2	0.003
**C-reactive protein [mg/dL]**	0.05 (0.03–0.10)	0.06 (0.03–0.12)	0.04 (0.03–0.07)	0.06 (0.03–0.11)	0.234
**Total cholesterol [mg/dL]**	191 ± 40	186 ± 34	192 ± 38	195 ± 47	0.669
**Triglycerides [mg/dL]**	128 (85–178)	125 (85–177)	111 (79–163)	130 (86–239)	0.509
**HDL cholesterol [mg/dL]**	54 ± 15	54 ± 14	57 ± 14	53 ± 16	0.610
**LDL cholesterol [mg/dL]**	101 ± 30	99 ± 27	102 ± 31	101 ± 33	0.935
**Corrected calcium [mg/dL]** **	9.2 ± 0.5	9.3 ± 0.6	9.4 ± 0.3	9.1 ± 0.6	0.137
**Phosphate [mg/dL]**	3.5 ± 0.7	3.4 ± 0.8	3.3 ± 0.5	3.8 ± 0.8	0.005
**Ferritin [ng/mL]**	76 (46–136)	78 (51–106)	61 (39–138)	80 (55–156)	0.607
***β***_**2**_ **-microglobulin [mg/L]**	3.2 (2.5–5.5)	2.5 (1.9–2.8)	3.1 (2.6–3.5)	5.8 (4.9–8.2)	<0.001
**Hemoglobin A1c [%]**	6.0 ± 0.7	5.9 ± 0.6	5.9 ± 0.5	6.1 ± 0.8	0.238
**Whole PTH [pg/mL]**	36 (26–53)	27 (22–33)	33 (25–43)	64 (45–102)	<0.001
**NT-proBNP [pg/mL]**	120 (56–253)	61 (31–107)	99 (48–204)	234 (127–578)	0.163
**eGFR [mL/min/1.73 m** ^**2**^ **]**	36.9 ± 15.2	53.2 ± 5.8	38.4 ± 4.5	20.0 ± 5.8	<0.001
**UPCR [g/g·creatinine]**	0.37 (0.09–1.64)	0.11 (0.06–0.50)	0.30 (0.13–1.40)	1.3 (0.4–3.6)	0.002
**Hemoglobin [g/dL]**	12.3 ± 1.5	12.9 ± 1.2	12.8 ± 1.4	11.4 ± 1.4	<0.001

* *P* value was calculated by chi-square test;

** Corrected calcium was calculated by the formula: (4.0 –serum albumin) + actual serum calcium.

### Clinical evaluation and laboratory measurements

All examinations were performed at the Medical Examination Center in Kyushu University Hospital without insurance. All of the patients underwent brain MRI scans. Clinical parameters were measured on the same day. Blood pressure in the brachial artery was measured in the sitting position after a 10-min rest. The height and weight of participants were measured, and their body mass index was calculated (kg/m^2^).

Blood samples were collected on the same day as undergoing MRI and were analyzed at the laboratory of Kyushu University Hospital, except for whole parathyroid hormone and N-terminal pro-brain natriuretic peptide (NT-proBNP), which were analyzed at a commercial laboratory (SRL Inc., Fukuoka, Japan). Serum chemistry values were measured using an auto-analyzer with standard procedures (Hitachi 911 Auto Analyzer; Hitachi Co. Ltd, Tokyo, Japan).

### Imaging data

**Fig 1:**
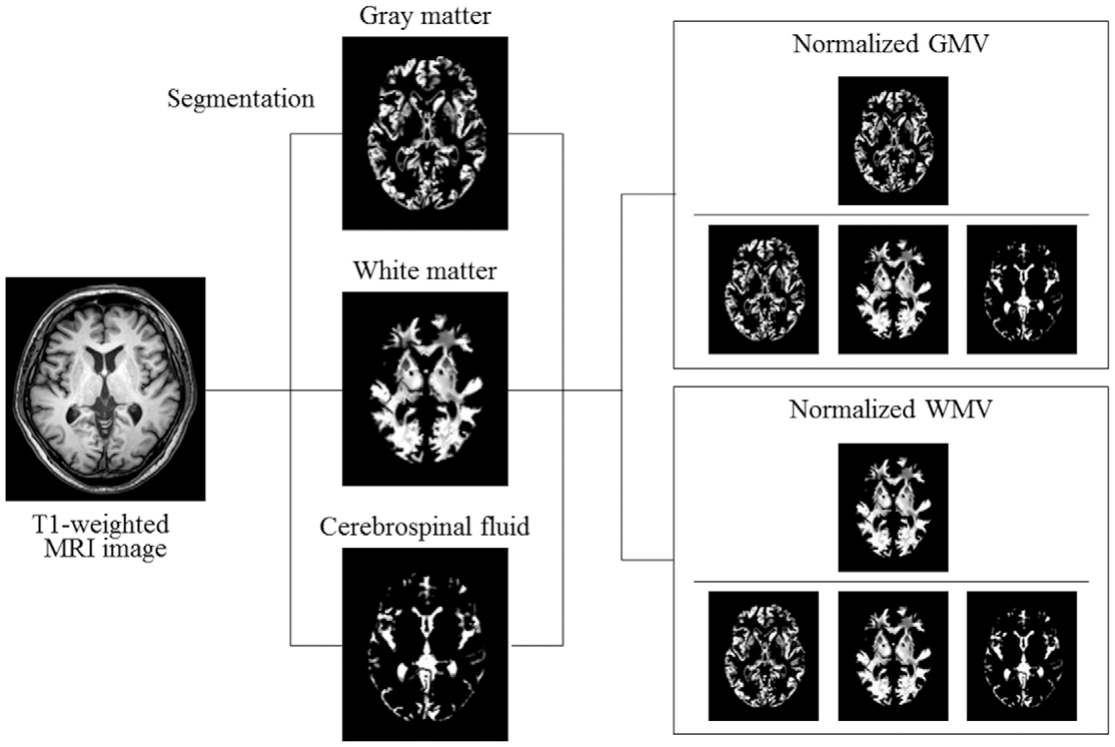
Segmentation of brain magnetic resonance imaging (MRI) and normalization of gray matter volume (GMV) and white matter volume (WMV). Representative axial brain image of T1-weighted MRI and segmented images of gray matter, white matter, and cerebrospinal fluid are shown. To normalize for head size variability, GMV and WMV were normalized by dividing by the total intracranial volume, calculated by adding GMV, WMV, and cerebrospinal fluid space volume.

Brain MRI was acquired from each subject using a 3.0 T Philips Achieva magnetic resonance scanner (Philips Health Care, Best, the Netherlands) at Kyushu University Hospital. No major hardware upgrades occurred during the study period. All of the patients were scanned with identical pulse sequences: 44 contiguous, 3.0-mm-thick axial planes of three-dimensional T1-weighted images (magnetization-prepared rapid acquisition of gradient echo: echo time, 3.7 ms; flip angle, 8; voxel size, 0.47 × 0.47 × 3 mm). The MRI imaging data were analyzed by a single investigator (H.Y.) who was blind to the clinical information as described previously [21]. We used Statistical Parametric Mapping 8 software (SPM8; Wellcome Department of Imaging Neuroscience, University College London, London, UK) to preprocess brain images. The segmentation algorithm from SPM8 was applied to every T1-weighted MRI scan to extract tissue maps corresponding to gray matter, white matter, and cerebrospinal fluid (Fig 1).

We applied these processes using the MATLAB file “cg_vbm_optimized” (http://dbm.neuro.uni-jena.de/vbm.html). The voxel values of each segmented image did not consist of binary (i.e., 0 or 1), but 256-level (i.e., between 0/255 and 255/255) signal intensities instead, according to their tissue probability. The linear-normalized, segmented images were restored to the native space, using the inverse normalization parameters calculated in normalizing each MR image to the Talairach space, to determine the volumes of each segment. The actual volumes of the entire normalized, segmented, and restored gray matter, white matter, and cerebral spinal fluid space images were determined automatically by summing voxel volumes multiplied by each voxel value and dividing by 255.

To normalize for head size variability, the GMV and white matter volume (WMV) were calculated as a percentage of the total intracranial volume, calculated by adding volumes of gray matter, white matter, and cerebrospinal fluid. These MRI measurements according to CKD stages are shown in Table 2.

**Table 2:** TMT Scores and MRI measurements of All Participants According to CKD Stages. Abbreviations: CSFV, cerebrospinal fluid volume; GMV, gray matter volume; TICV, total intracranial volume; TMT, Trail Making Test; WMV, white matter volume.

	All participants (*n* = 95)	CKD stage 3a (*n* = 34)	CKD stage 3b (*n* = 26)	CKD stages 4+5 (*n* = 35)	*P* for trend
**TMT-A**	41 ± 20	41 ± 19	36 ± 15	45 ± 22	0.382
**TMT-B**	106 ± 67	105 ± 61	90 ± 39	118 ± 86	0.398
**ΔTMT**	64 ± 53	63 ± 49	54 ± 28	73 ± 69	0.450
**GMV**	647 ± 50	654 ± 45	655 ± 53	635 ± 51	0.127
**WMV**	641 ± 39	634 ± 33	641 ± 44	647 ± 39	0.146
**CSFV**	315 ± 38	312 ± 37	312 ± 42	320 ± 37	0.388
**TICV**	1,603 ± 74	1,600 ± 66	1,608 ± 84	1,603 ± 76	0.864
**Normalized GMV**	40.4 ± 2.4	40.9 ± 2.1	40.7 ± 2.6	39.6 ± 2.5	0.035
**Normalized WMV**	40.0 ± 1.6	39.6 ± 1.6	39.8 ± 1.6	40.4 ± 1.6	0.054
**Normalized frontal GMV**	11.6 ± 0.8	11.8 ± 0.7	11.8 ± 0.8	11.3 ± 0.9	0.011
**Normalized parietal GMV**	5.1 ± 0.4	5.2 ± 0.4	5.1 ± 0.4	5.0 ± 0.4	0.020
**Normalized temporal GMV**	6.5 ± 0.5	6.6 ± 0.5	6.6 ± 0.5	6.3 ± 0.5	0.016
**Normalized occipital GMV**	4.1 ± 0.3	4.2 ± 0.4	4.1 ± 0.3	4.0 ± 0.3	0.024

### Trail Making Test

The TMT has been widely used as the test for a subject’s visual attention and task switching ability. Originally, it was part of the Army Individual Test Battery and subsequently was incorporated into the Halstead-Reitan Battery [22]. It consists of parts A and B (TMT-A and TMT-B). TMT-A requires an individual to connect randomly located numbers in numerical order as rapidly as possible, whereas TMT-B contains both numbers and letters, and the subject is required to connect the numbers and letters alternately. The score on each part of the TMT is the amount of time required to complete the task. The TMT-A score reflects visual search ability and motor skills, whereas the TMT-B score additionally reflects the ability for cognitive alternation [23,24].

The tests were conducted according to the procedure described by Hirota et al. [25] During the tests, the examiner corrected each error immediately. The time to complete each part of the TMT was recorded, and raw time scores (in seconds) were used as the dependent variables. TMT-A requires the individuals to draw lines to connect consecutively numbered circles (1–25) randomly arranged on a page, as fast as possible. TMT-B adds a measure of cognitive flexibility by asking the individual to connect the same number of circles in an alternating sequence of numbers and letters in the Japanese alphabet “hiragana”. The time limits for performing TMT-A and TMT-B were set at 180 s and 480 s, respectively.

The time difference between TMT-A and TMT-B (TMT-B minus TMT-A; ΔTMT) was also used as an assessment parameter so that we were able to control for the effect of motor speed on TMT performance and to evaluate executive function more accurately than simply using the performance of TMT-A or TMT-B alone. The TMT scores according to CKD stages are shown in Table 2.

### Statistical analyses

The Student’s *t*-test, Mann–Whitney test, and chi-square test were used, as appropriate, to describe the difference in baseline characteristics of the patients. We performed univariable and multivariable regression analyses to investigate the correlation between normalized GMV and TMT. We entered age, sex, diabetes mellitus, smoking habits, drinking habits, systolic blood pressure, past history of cardiovascular disease, education, hemoglobin, eGFR, and log-transformed urinary protein to creatinine ratio as covariates in the multivariable regression model by the forced entry method. We selected these covariates because they are considered to affect executive functioning. All statistical analyses were performed using JMP version 11.0 software (SAS Institute, Inc., Cary, NC, USA).

## Results

### Correlation of age with normalized GMV, but not normalized WMV

**Fig 2:**
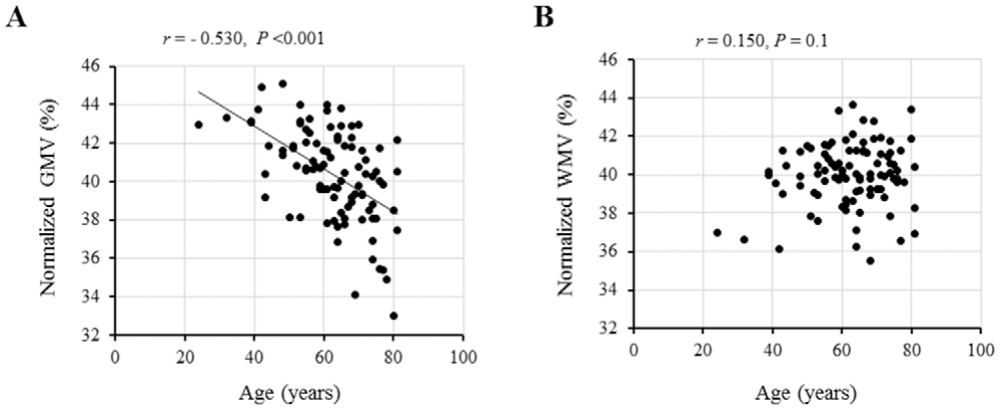
Correlations between age and normalized gray matter volume (GMV) and normalized white matter volume (WMV). A significant inverse correlation is found between age and normalized GMV (A), while no correlation is found between age and normalized WMV (B).

In the univariable regression analysis, normalized GMV, but not normalized WMV, was significantly correlated with age (Fig 2).

### No Correlation of eGFR with TMT

In the univariable and multivariable regression analysis, eGFR was not significantly correlated with any TMT scores (Table A in S1 File).

### Correlation of normalized GMV, but not normalized WMV, with TMT

**Fig 3:**
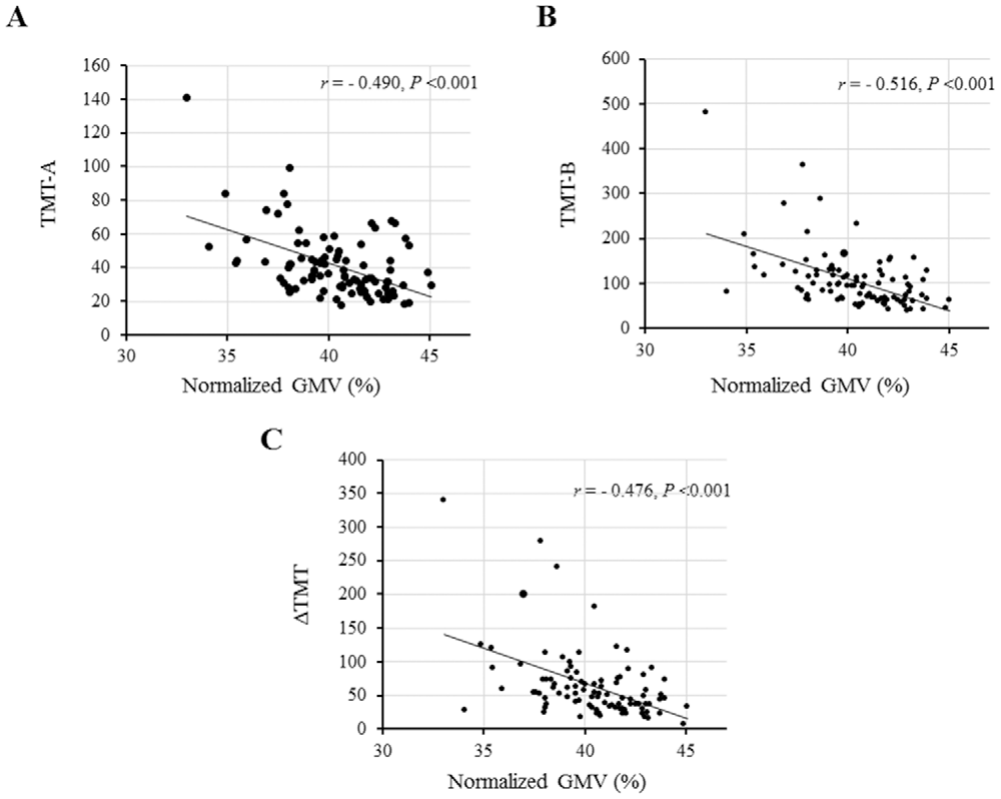
Correlations between normalized gray matter volume (GMV) and the scores (times in seconds) of Trail Making Test (TMT). Significant inverse correlations are found between normalized GMV and each score of TMT-A (A), TMT-B (B), and ΔTMT (C).

**Fig 4:**
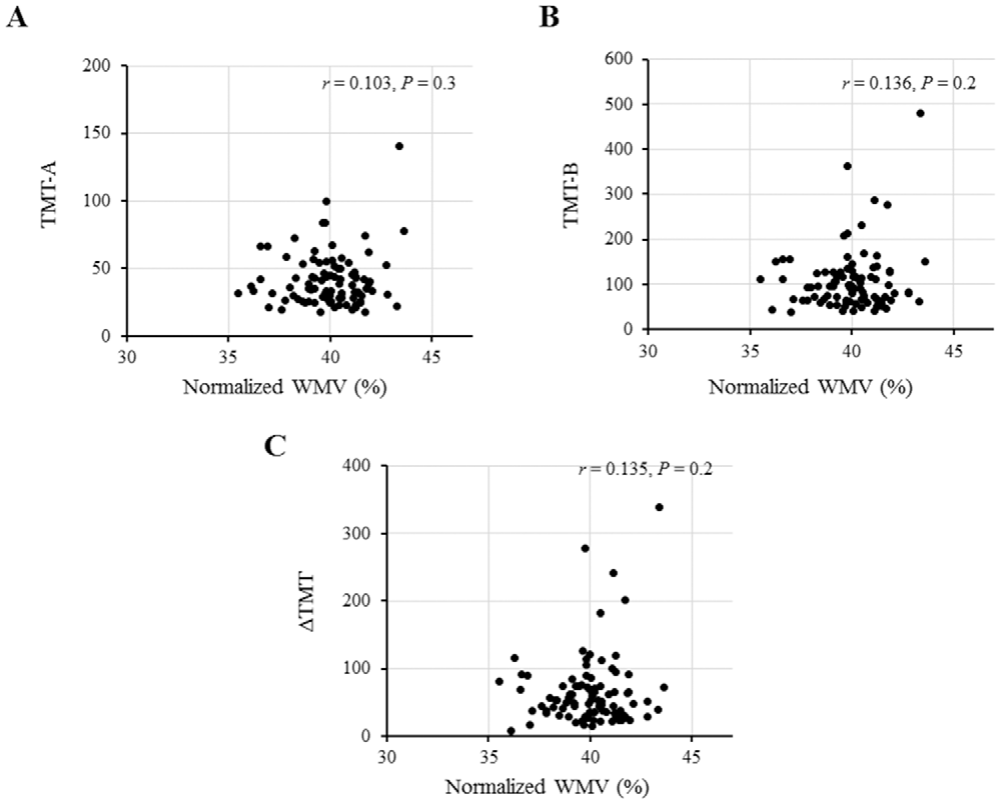
Correlations between normalized white matter volume (WMV) and the scores (times in seconds) of Trail Making Test (TMT). No correlations are found between normalized WMV and each score of TMT-A (A), TMT-B (B), and ΔTMT (C).

Next, we examined the correlation of normalized GMV and normalized WMV with TMT. In the univariable regression analysis, normalized GMV was significantly correlated with all scores of TMT-A, TMT-B, and ΔTMT (Fig 3) and these correlations were significant even after adjusting for relevant confounding factors (Table 3). These findings suggest that a decrease in normalized GMV results in longer TMT performance times. However, there was no correlation between normalized WMV and TMT scores (Fig 4).

**Table 3:** Univariable and Multivariable-Adjusted Regression Analyses of Correlation between Normalized Whole-Brain GMV and TMT Scores in All Participants. Model I: Multivariable analysis adjusted for sex and age. Model II: Model I + diabetes mellitus, estimated glomerular filtration rate, and education. Model III: Model II + systolic blood pressure, smoking habits, drinking habits, hemoglobin, previous history of cardiovascular disease, and log-transformed urinary protein to creatinine ratio. Abbreviations: TMT, Trail Making Test.

		TMT-A	TMT-B	ΔTMT
**Univariable analysis**	***Standardized β***	- 0.490	- 0.516	- 0.476
	***P***	<0.001	<0.001	<0.001
**Model I**	***Standardized β***	- 0.442	- 0.467	- 0.432
	***P***	<0.001	<0.001	<0.001
**Model II**	***Standardized β***	- 0.394	- 0.423	- 0.393
	***P***	0.002	<0.001	0.003
**Model III**	***Standardized β***	- 0.349	- 0.362	- 0.332
	***P***	0.012	0.006	0.013

### Correlation between normalized GMV and TMT stratified by eGFR (<45 vs. ≥45 mL/min/1.73 m^2^ and age (<65 vs. ≥65 years)

When stratified by eGFR (<45 vs. ≥45 mL/min/1.73 m^2^), multivariable associations were observed in participants with eGFR <45 mL/min/1.73 m^2^, but not in those with eGFR ≥45 mL/min/1.73 m^2^, although univariable associations were observed in both participants (Table 4). Similarly, when stratified by age (<65 vs. ≥65 years), multivariable associations were observed in participants ≥65 years, but not in participants <65 years, although univariable associations were observed in both participants (Table 5).

**Table 4:** Univariable and Multivariable-Adjusted Regression Analyses of Correlation between Normalized Whole-Brain GMV and TMT Scores in Participants Stratified by eGFR (<45 vs. ≥45 mL/min/1.73 m^2^). Model I: Multivariable analysis adjusted for sex and age. Model II: Model I + diabetes mellitus, estimated glomerular filtration rate, and education. Model III: Model II + systolic blood pressure, smoking habits, drinking habits, hemoglobin, previous history of cardiovascular disease, and log-transformed urinary protein to creatinine ratio. Abbreviations: eGFR, estimated glomerular filtration rate; TMT, Trail Making Test.

		TMT-A		TMT-B		ΔTMT	
		eGFR <45 mL/min/1.73 m^2^ (*n* = 61)	eGFR ≥45 mL/min/1.73 m^2^ (*n* = 34)	eGFR <45 mL/min/1.73 m^2^ (*n* = 61)	eGFR ≥45 mL/min/1.73 m^2^ (*n* = 34)	eGFR <45 mL/min/1.73 m^2^ (*n* = 61)	eGFR ≥45 mL/min/1.73 m^2^ (*n* = 34)
**Univariable analysis**	***Standardized β***	- 0.549	- 0.368	- 0.576	- 0.375	- 0.537	- 0.332
	***P***	<0.001	0.032	<0.001	0.029	<0.001	0.055
	***P* for interaction**	0.569		0.401		0.401	
**Model I**	***Standardized β***	- 0.477	- 0.477	- 0.512	- 0.460	- 0.482	- 0.398
	***P***	<0.001	0.068	<0.001	0.079	<0.001	0.134
	***P* for interaction**	0.383		0.244		0.244	
**Model II**	***Standardized β***	- 0.448	- 0.280	- 0.514	- 0.106	- 0.495	- 0.026
	***P***	0.002	0.325	<0.001	0.672	<0.001	0.917
	***P* for interaction**	0.537		0.358		0.358	
**Model III**	***Standardized β***	- 0.407	- 0.200	- 0.472	- 0.011	- 0.456	0.063
	***P***	0.016	0.557	0.003	0.968	0.005	0.827
	***P* for interaction**	0.574		0.400		0.401	

**Table 5:** Univariable and Multivariable-Adjusted Regression Analyses of Correlation between Normalized Whole-Brain GMV and TMT Scores in Participants Stratified by Age (<65 vs. ≥65 years). Model I: Multivariable analysis adjusted for sex and age. Model II: Model I + diabetes mellitus, estimated glomerular filtration rate, and education. Model III: Model II + systolic blood pressure, smoking habits, drinking habits, hemoglobin, previous history of cardiovascular disease, and log-transformed urinary protein to creatinine ratio. Abbreviation: GMV, gray matter volume; TMT, Trail Making Test.

		TMT-A		TMT-B		ΔTMT	
		Age <65 years (*n* = 51)	Age ≥65 years (*n* = 44)	Age <65 years (*n* = 51)	Age ≥65 years (*n* = 44)	Age <65 years (*n* = 51)	Age ≥65 years (*n* = 44)
**Univariable analysis**	***Standardized β***	- 0.256	- 0.500	- 0.389	- 0.472	- 0.398	- 0.412
	***P***	0.070	<0.001	0.005	0.001	0.004	0.006
	***P* for interaction**	0.112		0.404		0.644	
**Model I**	***Standardized β***	- 0.291	- 0.422	- 0.346	- 0.462	- 0.331	- 0.427
	***P***	0.097	0.004	0.040	0.003	0.048	0.009
	***P* for interaction**	0.115		0.386		0.615	
**Model II**	***Standardized β***	0.018	- 0.419	- 0.200	- 0.484	- 0.259	- 0.455
	***P***	0.933	0.006	0.315	0.002	0.195	0.005
	***P* for interaction**	0.053		0.221		0.418	
**Model III**	***Standardized β***	0.079	- 0.397	- 0.153	- 0.370	- 0.221	- 0.321
	***P***	0.729	0.041	0.467	0.054	0.287	0.101
	***P* for interaction**	0.066		0.254		0.462	

### Correlation between normalized regional GMV and TMT

Executive function, related to frontal lobe function, is thought to be characteristically impaired in patients with CKD. Thus, we examined the correlation between normalized regional GMV and TMT. The results showed that normalized frontal and temporal GMVs were correlated with all scores of TMT-A, TMT-B, and ΔTMT and that these correlations were significant even after adjusting for relevant confounding factors (Table 6). Normalized parietal GMV was also correlated with TMT in the univariable regression analysis, but the significant correlation disappeared after multivariable adjustment (Table 6). Normalized occipital GMV was not correlated with any scores of TMT-A, TMT-B, and ΔTMT both in the univariable and multivariable regression analyses (Table 6).

**Table 6:** Univariable and Multivariable-Adjusted Regression Analyses of Correlation between Normalized Regional GMV and TMT. Abbreviations: GMV, gray matter volume; TMT, Trail Making Test.

		TMT-A	TMT-B	ΔTMT
**Normalized Frontal GMV**				
**Univariable analysis**	***Standardized β***	- 0.436	- 0.468	- 0.436
	***P***	<0.001	<0.001	<0.001
**Multivariable analysis** *	***Standardized β***	- 0.279	- 0.287	- 0.263
	***P***	0.03	0.02	0.03
**Normalized Temporal GMV**				
**Univariable analysis**	***Standardized β***	- 0.498	- 0.498	- 0.527
	***P***	<0.001	<0.001	<0.001
**Multivariable analysis** *	***Standardized β***	- 0.352	- 0.396	- 0.375
	***P***	0.005	<0.001	0.002
**Normalized Parietal GMV**				
**Univariable analysis**	***Standardized β***	- 0.283	- 0.333	- 0.320
	***P***	0.005	0.001	0.002
**Multivariable analysis** *	***Standardized β***	- 0.105	- 0.141	- 0.141
	***P***	0.4	0.2	0.2
**Normalized Occipital GMV**				
**Univariable analysis**	***Standardized β***	- 0.137	- 0.164	- 0.159
	***P***	0.2	0.1	0.1
**Multivariable analysis** *	***Standardized β***	0.003	- 0.009	0.060
	***P***	0.9	0.9	0.6

* Multivariable adjustment was performed for age, gender, diabetes mellitus, systolic blood pressure, smoking habits, drinking habits, education, hemoglobin, estimated glomerular filtration rate, and log-transformed urinary protein to creatinine ratio.

### Risk factors for decline in normalized GMV

We examined the risk factors of normalized GMV decline in patients with CKD stages 3–5. Covariates of *P* <0.1 in the univariable analysis were included in the multivariable analysis. Serum urea nitrogen, creatinine, and log-transformed *β*_2_-microglobulin levels were not included because of multi-collinearity with eGFR. As a result, age, male, diabetes, higher NT-proBNP, and lower hemoglobin were correlated with lower normalized GMV in the multivariable regression analysis (Table 7). On the other hand, there was no multivariable association of any of these factors with normalized WMV (Table B in S1 File).

**Table 7:** Univariable and Multivariable-Adjusted Regression Analyses for Normalized GMV. Abbreviations: CRP, C-reactive protein; CVD, cardiovascular disease; eGFR, estimated glomerular filtration rate; ESA, erythropoiesis-stimulating agent; GMV, gray matter volume; HDL, high-density lipoprotein; LDL, low-density lipoprotein; NT-proBNP, N-terminal pro-brain natriuretic peptide; PTH, parathyroid hormone; RAAS, renin-angiotensin-aldosterone system; UPCR, urinary protein to creatinine ratio.

	Univariable analysis		Multivariable analysis *	
	*Standardized β*	*P*	*Standardized β*	*P*
**Age**	- 0.530	<0.001	- 0.346	<0.001
**Male gender**	- 0.384	<0.001	- 0.306	0.003
**Diabetes mellitus**	- 0.394	<0.001	- 0.248	0.004
**Smoking habits**	- 0.276	0.007	0.026	0.790
**Daily alcohol consumption**	0.063	0.547		
**Previous history of CVD**	- 0.293	0.004	- 0.118	0.141
**Education of more than 12 years**	0.145	0.162		
**Body mass index**	- 0.068	0.514		
**Systolic blood pressure**	- 0.145	0.160		
**Diastolic blood pressure**	0.165	0.110		
**Use of RAAS inhibitors**	- 0.194	0.060	0.169	0.068
**Use of calcium antagonists**	0.209	0.042	- 0.020	0.817
**Use of statins**	0.100	0.335		
**Use of ESAs**	0.081	0.437		
**Total protein**	0.061	0.559		
**Albumin**	0.177	0.086	0.027	0.736
**Serum urea nitrogen**	- 0.269	0.008		
**Creatinine**	- 0.253	0.013		
**Uric acid**	0.088	0.398		
**Log-transformed CRP**	- 0.114	0.271		
**Total cholesterol**	0.120	0.247		
**Log-transformed triglycerides**	0.056	0.452		
**HDL cholesterol**	0.036	0.732		
**LDL cholesterol**	0.167	0.105		
**Calcium**	0.111	0.284		
**Phosphate**	- 0.038	0.716		
**Log-transformed ferritin**	- 0.165	0.110		
**Log-transformed *β***_**2**_ **-microglobulin**	- 0.262	0.010		
**Hemoglobin A1c**	- 0.081	0.437		
**Log-transformed whole PTH**	- 0.304	0.003	- 0.064	0.607
**Log-transformed NT-proBNP**	- 0.406	<0.001	- 0.142	0.192
**eGFR**	0.206	0.045	- 0.133	0.297
**Log-transformed UPCR**	- 0.039	0.709		
**Hemoglobin**	0.232	0.023	0.181	0.049

* Covariates of *P* <0.1 in the univariable analysis were included in the multivariable analysis, whereas serum urea nitrogen, creatinine, and log-transformed *β*_2_-microglobulin were not included because of multi-collinearity with estimated glomerular filtration rate.

## Discussion

The present study showed a negative correlation between normalized GMV and TMT in patients with CKD stages 3–5, even after adjustment for relevant risk factors. This result suggests that atrophy of brain gray matter could be an independent risk factor for frontal lobe executive dysfunction.

First of all, the accuracy of the measurements of brain volume using MRI is considered the most important issue in the present study, in which, our data showed that normalized GMV, but not normalized WMV, was significantly correlated with age. This finding was similar to previous reports by Taki et al. and our studies [21,26], suggesting that our measurements of normalized GMV and WMV using MRI were reliable and accurate.

Recently, several studies have examined alterations in brain volume and cognitive function, and the results showed an association between decreased brain volume and cognitive impairment in patients with diabetes [14–16], Parkinson’s disease [17], Alzheimer’s disease [18], and in the general elderly population with age-related cerebral small vessel disease [19]. However, not all studies observed these associations, even with large sample sizes (*n* >100) [27]. In CKD, Zhang et al. [20] reported that neuropsychological test scores correlated with some decreased gray matter volume in ESRD patients, while no correlation was found between WMV and any neuropsychological test scores in ESRD patients. They also found predominantly decreased gray matter volume in ESRD patients, which was correlated with neurocognitive dysfunction, and speculated that the volume loss of this gray matter is correlated with the development of neurocognitive dysfunction in patients with ESRD [20]. These findings are in agreement with the results of the present study.

In our study, the correlation between normalized regional GMV and TMT shows that normalized frontal and temporal GMV, but not parietal or occipital GMV, are significantly inversely correlated to TMT-A, TMT-B, and ΔTMT scores using multivariable regression analysis. This result is considered to be reasonable because TMT is an indicator of frontal lobe function. Hypoperfusion in the frontal lobe has been observed in HD patients in one previous study using single photon emission tomography [28]. These findings can partially explain ESRD patients’ neurocognitive dysfunction, such as attention, mental processing, memory, and perceptual-motor difficulties [4,29].

In regard to the mechanism of brain atrophy and executive dysfunction in CKD patients, we previously reported a 3-year prospective study in which dialysis-related hypotension appeared to play a role in causing progressive frontal lobe atrophy in chronic HD patients [30]. However, the subjects in our study are patients with NDD-CKD in whom a rapid decline in blood pressure and brain blood flow cannot occur. Nevertheless, it has been reported that brain volume is decreased in NDD-CKD patients compared with those without CKD. Yakushiji et al. [10] showed that decreased GFR was significantly correlated with brain atrophy and this correlation remained significant even after adjusting for confounding factors such as age, sex, hypertension, white matter hyperintensities, and the presence of lacunae. Thus, we examined the risk factors of normalized GMV in patients with CKD stages 3–5 and found that age, male, diabetes, higher NT-proBNP, and lower hemoglobin were correlated with lower normalized GMV in the multivariable regression analysis. This result suggests that aging- and diabetes-induced atherosclerotic vascular change and endothelial dysfunction, and anemia-induced chronic oxygen deficiency in the brain might accelerate brain atrophy. This is consistent with the findings of previous reports in which age, diabetes, and anemia are suggested as causes of brain atrophy [26,31,32].

In particular, the involvement of anemia in the brain atrophy is of great interest. That is, we previously investigated the effects of anemia correction with recombinant human erythropoietin on cerebral blood flow and oxygen metabolism in HD patients, and showed that regional cerebral metabolic rate for oxygen (rCMRO_2_) significantly increased in the frontal cerebral cortex, but not in the other cerebral cortices, deep gray matter, white matter, and cerebellum, after the correction of anemia [33]. Marsh et al. [34] have also reported that the P_3_-wave amplitude of the brain evoked potential increased after recombinant human erythropoietin, and its increase was the largest on the frontal lobe. From these findings, it is suggested that anemia correction might be the most effective strategy for prevention of brain atrophy and executive dysfunction in patients with CKD.

More recently, we reported in the cross-sectional and longitudinal study that the decline in normalized GMV is more rapid in patients with PD than in patients with NDD-CKD [21]. Although we could not elucidate the cause of rapid brain atrophy in PD patients in that report, we suspect the involvement of uremic toxin and oxidative stress in rapid brain atrophy in advanced CKD patients. A recent report by Zhang et al. [20] also demonstrated that high serum urea level can be a risk factor for development of mild cognitive impairment in ESRD patients. Our data also showed that serum urea nitrogen was significantly inversely correlated with normalized GMV in the univariable analysis (Table 4), although the statistical significance disappeared when the variable was entered to the multivariable model instead of eGFR (data not shown). This is in accordance with our experimental reports published recently, in which an impairment of spatial working memory due to accumulation of 8-hydroxy-2'-deoxyguanosine and increased numbers of pyknotic neuronal cells in the hippocampus of subtotal nephrectomized CKD mice was inhibited by tempol or telmisartan through their antioxidative effect [35,36]. From these findings, we speculate the potential mechanism of brain atrophy and executive dysfunction in CKD patients as follows: In CKD, uremic toxin-induced oxidative stress induces neuronal cell damage, followed by neuronal cell death and decrease in gray matter volume, resulting in executive dysfunction.

Murea et al. [37] recently examined the relationships between mild-to-moderate CKD and brain morphology and cognitive performance in 478 participants with eGFR ≥45 mL/min/1.73 m^2^ and demonstrated that kidney function parameters were not significantly associated with striking changes in brain structure or cognitive performance in those with mild CKD. The findings in their study were consistent with the observations in our study, although participants with eGFR <45 mL/min/1.73 m^2^ was excluded, unlike our study. Thus, we stratified the participants by eGFR (<45 vs. ≥45 mL/min/1.73 m^2^) and examined the associations between normalized GMV and TMT scores. Then, multivariable associations were observed in participants with eGFR <45 mL/min/1.73 m^2^, but not in participants with eGFR ≥45 mL/min/1.73 m^2^, whereas univariable associations were observed in both participants. According to this finding, it is considered that the correlation of brain atrophy with executive dysfunction is more robust in patients with severe renal dysfunction.

Furthermore, when stratified by age (<65 vs. ≥65 years), multivariable associations were observed in participants ≥65 years, but not in participants <65 years, whereas univariable associations were observed in both participants. We suppose that the smaller normalized GMV (more severe brain atrophy) and the higher TMT scores (more severe executive dysfunction) in the elderly compared with younger participants might attribute to the more robust association between them in the elderly probably due to the threshold effect reported in the neuropsychological correlates of white-matter lesions in healthy elderly subjects [38].

There are several limitations in the present study. First, the number of subjects is relatively small. Thus, multivariate adjustment might not be appropriate for the sub-groups analysis with a few sample. Second, the cross-sectional study design limits the interpretation of causality between brain atrophy and executive dysfunction. However, we consider that this causality is plausible from the common sense view that executive dysfunction could not induce brain atrophy, although the opposite could be possible. Third, single measurements of clinical parameters and laboratory data could be inaccurate. Fourth, although the involvement of imbalanced autonomic nervous system in the impaired cognitive function has been reported in many neurodegenerative diseases like Alzheimer [39], we could not examine the relationship between acetylcholine-mediated neurotransmission and cognitive function because we had not any data regarding autonomic nerve function. Fifth, only executive function rather than general and specific cognitive function was measured, which decrease the clinical value of measuring the brain volume for patients with varied extent of cognitive dysfunction on different aspects.

The strong point of this study is that, at least to our knowledge, it offers the first report of the correlation between brain atrophy and executive function in CKD patients. This study is thought to be particularly worthy because it shows the possible impact of frontal lobe atrophy on frontal lobe dysfunction in NDD-CKD patients. Furthermore, the quantification and measurement of normalized GMV is very accurate and reputable.

In conclusion, the present study demonstrates the close correlation between normalized GMV and executive function as determined by TMT, especially in the frontal and temporal regions. Therefore, we must keep in mind the benefits of preventing a decrease in normalized GMV in maintaining executive function, although how best to prevent brain atrophy remains unclear. However, several limitations preclude from a convincing conclusion resulted from this study. Thus, further longitudinal study is needed to elucidate the causative factors inducing brain atrophy and how to prevent the associated risk factors.

## Supporting Information

S1 File **Table A.** Univariable and Multivariable-Adjusted Regression Analyses for TMT Scores; **Table B.** Univariable and Multivariable-Adjusted Regression Analyses for Normalized WMV.
(DOCX)
