# Supplementary material for: Clinical Significance of Fronto-Temporal Gray Matter Atrophy in Executive Dysfunction in Patients with Chronic Kidney Disease: The VCOHP Study
Source: PLoS One. 2015 Dec 3;10(12):e0143706. doi: 10.1371/journal.pone.0143706 (PMC4669129; doi:10.1371/journal.pone.0143706)
Supplement: S1 File — Table A. Univariable and Multivariable-Adjusted Regression Analyses for TMT Scores; Table B. Univariable and Multivariable-Adjusted Regression Analyses for Normalized WMV. (DOCX) [file pone.0143706.s001.docx]

# S1 File.

# Table A. Univariable and Multivariable-Adjusted Regression Analyses for TMT Scores

|  | **TMT-A** | | | | **TMT-B** | | | | **ΔTMT** | | | |  |
| --- | --- | --- | --- | --- | --- | --- | --- | --- | --- | --- | --- | --- | --- |
|  | **Univariable** | | **Multivariable *** | | **Univariable** | | **Multivariable *** | | **Univariable** | | **Multivariable *** | |  |
|  | ***β* ^#^** | ***P*** | ***β* ^#^** | ***P*** | ***β* ^#^** | ***P*** | ***β* ^#^** | ***P*** | ***β* ^#^** | ***P*** | ***β* ^#^** | ***P*** | |
| **Age** | 0.390 | <0.001 | 0.243 | 0.014 | 0.406 | <0.001 | 0.262 | 0.007 | 0.373 | <0.001 | 0.230 | 0.019 | |
| **Sex, male** | 0.082 | 0.427 |  |  | 0.090 | 0.384 |  |  | 0.085 | 0.414 |  |  | |
| **Diabetes mellitus** | 0.239 | 0.019 | 0.135 | 0.179 | 0.225 | 0.028 | 0.159 | 0.102 | 0.198 | 0.054 | 0.129 | 0.196 | |
| **Smoking habits** | - 0.021 | 0.840 |  |  | 0.031 | 0.768 |  |  | 0.047 | 0.654 |  |  | |
| **Daily alcohol consumption** | - 0.036 | 0.727 |  |  | - 0.099 | 0.340 |  |  | - 0.113 | 0.277 |  |  | |
| **Previous history of CVD** | 0.224 | 0.029 | 0.162 | 0.089 | 0.049 | 0.634 |  |  | - 0.019 | 0.855 |  |  | |
| **Education of more than 12 years** | - 0.166 | 0.107 |  |  | - 0.212 | 0.039 | - 0.096 | 0.299 | - 0.209 | 0.042 | - 0.092 | 0.330 | |
| **Body mass index** | 0.014 | 0.891 |  |  | 0.077 | 0.460 |  |  | 0.092 | 0.374 |  |  | |
| **Systolic blood pressure** | 0.127 | 0.222 |  |  | 0.226 | 0.027 | 0.014 | 0.886 | 0.241 | 0.018 | 0.055 | 0.583 | |
| **Diastolic blood pressure** | - 0.154 | 0.135 |  |  | - 0.122 | 0.238 |  |  | - 0.099 | 0.340 |  |  | |
| **Use of RAAS inhibitors** | - 0.175 | 0.090 | - 0.069 | 0.520 | - 0.215 | 0.036 | - 0.040 | 0.687 | -0.210 | 0.041 | - 0.035 | 0.736 | |
| **Use of calcium antagonists** | 0.187 | 0.070 | 0.081 | 0.431 | 0.135 | 0.193 |  |  | 0.103 | 0.320 |  |  | |
| **Use of statins** | 0.027 | 0.798 |  |  | 0.002 | 0.987 |  |  | -0.007 | 0.943 |  |  | |
| **Use of ESAs** | 0.018 | 0.864 |  |  | 0.031 | 0.767 |  |  | 0.033 | 0.754 |  |  | |
| **Total protein** | 0.053 | 0.612 |  |  | 0.063 | 0.545 |  |  | 0.061 | 0.559 |  |  | |
| **Albumin** | - 0.068 | 0.512 |  |  | - 0.069 | 0.508 |  |  | - 0.063 | 0.547 |  |  | |
| **Serum urea nitrogen** | 0.197 | 0.056 |  |  | 0.246 | 0.016 |  |  | 0.241 | 0.019 |  |  | |
| **Creatinine** | 0.129 | 0.212 |  |  | 0.192 | 0.062 |  |  | 0.197 | 0.055 |  |  | |
| **Uric acid** | - 0.072 | 0.489 |  |  | - 0.060 | 0.565 |  |  | - 0.050 | 0.632 |  |  | |
| **Log-transformed CRP** | 0.133 | 0.198 |  |  | 0.117 | 0.260 |  |  | 0.099 | 0.338 |  |  | |
| **Total cholesterol** | - 0.059 | 0.571 |  |  | - 0.097 | 0.350 |  |  | - 0.102 | 0.327 |  |  | |
| **Log-transformed triglycerides** | 0.053 | 0.607 |  |  | 0.048 | 0.641 |  |  | 0.042 | 0.686 |  |  | |
| **HDL cholesterol** | - 0.115 | 0.266 |  |  | - 0.143 | 0.166 |  |  | - 0.140 | 0.175 |  |  | |
| **LDL cholesterol** | - 0.089 | 0.391 |  |  | - 0.137 | 0.187 |  |  | - 0.141 | 0.172 |  |  | |
| **Corrected calcium** | - 0.103 | 0.321 |  |  | - 0.207 | 0.044 | - 0.107 | 0.257 | - 0.225 | 0.028 | - 0.138 | 0.155 | |
| **Phosphate** | 0.057 | 0.580 |  |  | 0.128 | 0.215 |  |  | 0.142 | 0.169 |  |  | |
| **Log-transformed ferritin** | 0.129 | 0.212 |  |  | 0.075 | 0.468 |  |  | 0.048 | 0.641 |  |  | |
| **Log-transformed *β*_2_-microglobulin** | 0.176 | 0.087 |  |  | 0.198 | 0.054 |  |  | 0.188 | 0.068 |  |  | |
| **Hemoglobin A1c** | - 0.062 | 0.553 |  |  | - 0.068 | 0.511 |  |  | - 0.064 | 0.537 |  |  | |
| **Log-transformed whole PTH** | 0.106 | 0.312 |  |  | 0.046 | 0.664 |  |  | 0.019 | 0.855 |  |  | |
| **Log-transformed NT-proBNP** | 0.364 | <0.001 | 0.344 | 0.006 | 0.416 | <0.001 | 0.388 | 0.002 | 0.396 | <0.001 | 0.360 | 0.005 | |
| **eGFR** | - 0.077 | 0.457 | 0.187 | 0.143 | - 0.067 | 0.516 | 0.187 | 0.138 | - 0.057 | 0.581 | 0.179 | 0.165 | |
| **Log-transformed UPCR** | - 0.060 | 0.565 |  |  | - 0.115 | 0.267 |  |  | - 0.124 | 0.230 |  |  | |
| **Hemoglobin** | - 0.117 | 0.259 |  |  | - 0.114 | 0.271 |  |  | - 0.102 | 0.325 |  |  | |

**^#^** Standardized *β*

* Covariates of *P* <0.1 in the univariable analysis and eGFR were included in the multivariable analysis, whereas serum urea nitrogen, creatinine, and log-transformed *β*_2_-microglobulin were not included because of multi-collinearity with eGFR.

Abbreviations: CRP, C-reactive protein; CVD, cardiovascular disease; eGFR, estimated glomerular filtration rate; ESA, erythropoiesis-stimulating agent; HDL, high-density lipoprotein; LDL, low-density lipoprotein; NT-proBNP, N-terminal pro-brain natriuretic peptide; PTH, parathyroid hormone; RAAS, renin-angiotensin-aldosterone system; TMT, Trail Making Test; UPCR, urinary protein to creatinine ratio.

# Table B. Univariable and Multivariable-Adjusted Regression Analyses for Normalized WMV

|  | **Univariable analysis** | | **Multivariable analysis *** | |
| --- | --- | --- | --- | --- |
|  | ***Standardized β*** | ***P*** | ***Standardized β*** | ***P*** |
| **Age** | 0.150 | 0.146 |  |  |
| **Sex, male** | 0.094 | 0.366 |  |  |
| **Diabetes mellitus** | 0.224 | 0.029 | 0.179 | 0.105 |
| **Smoking habits** | 0.226 | 0.028 | 0.083 | 0.464 |
| **Daily alcohol consumption** | 0.034 | 0.745 |  |  |
| **Previous history of CVD** | 0.163 | 0.116 |  |  |
| **Education of more than 12 years** | 0.067 | 0.521 |  |  |
| **Body mass index** | - 0.087 | 0.400 |  |  |
| **Systolic blood pressure** | 0.112 | 0.278 |  |  |
| **Diastolic blood pressure** | 0.086 | 0.406 |  |  |
| **Use of RAAS inhibitors** | 0.096 | 0.354 |  |  |
| **Use of calcium antagonists** | - 0.095 | 0.359 |  |  |
| **Use of statins** | - 0.012 | 0.905 |  |  |
| **Use of ESAs** | - 0.028 | 0.788 |  |  |
| **Total protein** | - 0.125 | 0.227 |  |  |
| **Albumin** | - 0.044 | 0.672 |  |  |
| **Serum urea nitrogen** | 0.106 | 0.308 |  |  |
| **Creatinine** | 0.135 | 0.191 |  |  |
| **Uric acid** | - 0.050 | 0.627 |  |  |
| **Log-transformed CRP** | - 0.020 | 0.848 |  |  |
| **Total cholesterol** | - 0.145 | 0.161 |  |  |
| **Log-transformed triglycerides** | - 0.176 | 0.087 | - 0.178 | 0.096 |
| **HDL cholesterol** | 0.050 | 0.628 |  |  |
| **LDL cholesterol** | - 0.175 | 0.090 | - 0.099 | 0.342 |
| **Corrected calcium** | - 0.135 | 0.192 |  |  |
| **Phosphate** | - 0.006 | 0.956 |  |  |
| **Log-transformed ferritin** | 0.051 | 0.625 |  |  |
| **Log-transformed *β*_2_-microglobulin** | 0.051 | 0.623 |  |  |
| **Hemoglobin A1c** | 0.072 | 0.489 |  |  |
| **Log-transformed whole PTH** | 0.209 | 0.044 | 0.055 | 0.714 |
| **Log-transformed NT-proBNP** | 0.219 | 0.033 | 0.065 | 0.623 |
| **eGFR** | - 0.188 | 0.068 | 0.035 | 0.821 |
| **Log-transformed UPCR** | - 0.047 | 0.651 |  |  |
| **Hemoglobin** | - 0.273 | 0.007 | - 0.150 | 0.219 |

* Covariates of *P* <0.1 in the univariable analysis were included in the multivariable analysis.

Abbreviations: CRP, C-reactive protein; CVD, cardiovascular disease; eGFR, estimated glomerular filtration rate; ESA, erythropoiesis-stimulating agent; HDL, high-density lipoprotein; LDL, low-density lipoprotein; NT-proBNP, N-terminal pro-brain natriuretic peptide; PTH, parathyroid hormone; RAAS, renin-angiotensin-aldosterone system; UPCR, urinary protein to creatinine ratio, WMV, white matter volume.
